# Crucial Role of the C-Terminal Domain of Hfq Protein in Genomic Instability

**DOI:** 10.3390/microorganisms8101598

**Published:** 2020-10-17

**Authors:** Virali J. Parekh, Frank Wien, Wilfried Grange, Thomas A. De Long, Véronique Arluison, Richard R. Sinden

**Affiliations:** 1 Laboratory of DNA Structure and Mutagenesis, Department of Biology, Chemistry and Health Sciences, South Dakota School of Mines and Technology, Rapid City, SD 57701, USA; virali.parekh@mines.sdsmt.edu (V.J.P.); thomas.delong@mines.sdsmt.edu (T.A.D.L.); 2 Synchrotron SOLEIL, 91192 Gif-sur-Yvette, France; frank.wien@synchrotron-soleil.fr; 3 Institut de Physique et Chimie des Matériaux de Strasbourg (IPCMS), Département d’Optique ultrarapide et de Nanophotonique (DON), 23, rue du Loess, BP 43, CEDEX 2, 67034 Strasbourg, France; wilfried.grange@u-paris.fr; 4 UFR Sciences du vivant–Université de Paris, F-75006 Paris, France; 5 Laboratoire Léon Brillouin (LLB), CEA, CNRS UMR12, Université Paris Saclay, CEA Saclay, 91191 Gif-sur-Yvette, France

**Keywords:** genomic instability, quadruplex, DNA-directed mutagenesis, nucleoid, bacterial chromatin

## Abstract

G-rich DNA repeats that can form G-quadruplex structures are prevalent in bacterial genomes and are frequently associated with regulatory regions of genes involved in virulence, antigenic variation, and antibiotic resistance. These sequences are also inherently mutagenic and can lead to changes affecting cell survival and adaptation. Transcription of the G-quadruplex-forming repeat (G_3_T)_n_ in *E. coli*, when mRNA comprised the G-rich strand, promotes G-quadruplex formation in DNA and increases rates of deletion of G-quadruplex-forming sequences. The genomic instability of G-quadruplex repeats may be a source of genetic variability that can influence alterations and evolution of bacteria. The DNA chaperone Hfq is involved in the genetic instability of these G-quadruplex sequences. Inactivation of the *hfq* gene decreases the genetic instability of G-quadruplex, demonstrating that the genomic instability of this regulatory element can be influenced by the *E. coli* highly pleiotropic Hfq protein, which is involved in small noncoding RNA regulation pathways, and DNA organization and packaging. We have shown previously that the protein binds to and stabilizes these sequences, increasing rates of their genomic instability. Here, we extend this analysis to characterize the role of the C-terminal domain of Hfq protein in interaction with G-quadruplex structures. This allows to better understand the function of this specific region of the Hfq protein in genomic instability.

## 1. Introduction

G-quadruplex comprises a class of alternative DNA and RNA structures in which three or more guanine quadruplex rings containing Hoogsteen hydrogen bonds [1] stack into remarkably stable four-stranded structures. G-quadruplex structures form in both RNA and DNA with highly variable topology in which RNA or DNA strands can be arranged in parallel, antiparallel, or mixed orientations associated with various glycosidic configurations of guanines [2,3,4,5]. In DNA, the C-rich DNA strand complementary to G-quadruplex-forming sequences can form a four stranded i-motif stabilized by low pH [6], in which two tracts of cytosines form interdigitated C•C^+^ base pairs [7,8,9].

DNA sequences that can form G-quadruplex structures are widely found in many species and are common in many genomes [10]. In higher organisms, G-quadruplex/i-motif structures occur in telomeric repeats, immunoglobulin switch regions, oncogene promoters, and in 5’ untranslated regions near translation start sites [4,5,7,11,12]. These sequences also occur in bacterial genomes [13,14,15]. Evidence suggests the potential involvement of G-quadruplex structures in the regulation of gene expression in *E. coli* [13,14,16,17]. When formed in mRNA, G-quadruplex structures can influence bacterial message utilization [18,19,20,21]. Evidence also suggests the involvement of G-quadruplexes in antigenic variation in bacteria and gene silencing in human immunodeficiency virus and Epstein–Barr virus [22]. A further example in *Plasmodium falciparum* quadruplexes may regulate translation efficiency and influence a host immune response [23,24,25,26,27].

Many proteins, in many different species, have been identified binding to G-quadruplex structures. These include helicases [24,28,29,30,31,32] and other proteins involved in replication and DNA repair [33,34]. Structural proteins involved in DNA and chromosome organization necessarily bind to DNA and the binding of the *E. coli* Hfq protein to DNA has been extensively well characterized [35,36,37,38,39,40]. The Hfq protein controls many critical bacterial functions. Among these functions, most are related to RNA-binding properties and Hfq plays a crucial role in Gram-negative bacteria RNA metabolism. In particular, it facilitates the pairing of regulatory small non-coding RNA (sRNA) with target mRNA(s), allowing a regulation at the post-transcriptional level [41,42]. We have recently shown that Hfq binds to G-quadruplex DNA, and that binding in *E. coli* leads to a dramatic increase in the rate of mutation associated with G-quadruplex-forming sequences [43]. 

Structurally, Hfq belongs to the Sm protein family and forms a hexameric toroidal ring [41,44]. Ring formation requires the amino-terminal region of Hfq (~65 amino acid residues) formed by an antiparallel β-sheet and an N-terminal α-helix. Both faces of the toroidal hexamer can bind nucleic acid, but DNA is rather bound on the surface where the N-terminal α-helices are exposed [45]. Hfq also comprises a C-terminal region (CTR) of about 40 amino acid residues located outside of the Sm ring [46]. Although no atomic 3D structure is known for this CTR, it binds DNA and self-assembles into an amyloid-like structure [47,48,49]. This CTR region plays a major role in DNA bridging and compaction [36,38,39]. Furthermore, Hfq-CTR also mediates competition between sRNAs, offering a possibility of cycling between competing RNAs [50]. In this work, we investigate a new property of the amyloid-like region of Hfq, specifically, a role in G-quadruplex binding and stability.

## 2. Materials and Methods 

### 2.1. Bacterial Strains and Media 

Bacterial strains used include *E. coli* MG1655 (K-12 F^–^ λ^–^ *ilvG*^–^ *rfb-50 rph-1*) and MG1655 derivatives [51]. In MG1655 *hfq*-Cm^r^, the *hfq* gene was replaced with a cloned *hfq* gene construct with cassette containing the *hfq* gene and an adjacent chloramphenicol acetyltransferase (*CAT*) gene. This strain (*hfq*^+^) is used as the reference strain in this manuscript. In MG1655 HfqNTR72-Cm^r^ (corresponding to *∆CTR-Hfq* variant), the cassette containing a region of the gene encoding only the first 72 amino acids of Hfq was inserted (NTR72, hereafter referred to as ∆CTR) with the adjacent *CAT* gene. This construct results in the expression of the first 72 amino acids of Hfq only. In MG1655 ΔHfq::Cm^r^, the *CAT* gene cassette insertion results in Hfq inactivation [38]. 

Luria–Bertani broth (LB) [52] was supplemented with 30 μg/mL of ampicillin (Amp). LB plates for Luria–Delbrück fluctuation assays for chloramphenicol resistance (Cm^r^) contained 25 μg/mL of Cm. Selection of tetracycline resistant revertants (Tet^r^) utilized LB plates containing 25 μg/mL tetracycline for MG1655 derivatives.

### 2.2. Measurement of Tet^r^ Mutation Rates 

Plasmids pBR325 and pBR235 provide an excellent model for measuring rates of deletions of DNA sequences [43,52,53,54,55,56]. MG1655 derivatives were transformed with plasmids containing (G_3_T)_8_ inserted into the BamHI site in the *tet* gene [43]. These plasmids contain the (G_3_T)_8_ sequence cloned in the orientation in which the G-rich strand comprises the nontemplate (coding) strand, which can support G-quadruplex formation on transcription [43]. To ascertain potential differences in rates of instability when the G-rich strand comprises the leading or lagging strands of replication, the orientation of the unidirectional ColE1 replication origin and ampicillin gene is reversed in pBR325 and pBR235-based plasmids. 

Mutation rates were determined by Luria–Delbrück fluctuation assays [57]. An overnight culture, started from a single colony, was diluted to ~10^4^ cells/mL and eighteen parallel 5-mL cultures were then grown overnight to stationary phase. Viable cell counts were determined for six cultures by plating cell dilutions on plates containing ampicillin and chloramphenicol. All cultures were used to determine the number of Tet^r^ revertants, by plating all cells on LB + Tet plates. Control experiments confirmed a copy number of approximately 20 plasmids per cell for all the Hfq-Cm^r^ and HfqNTR72 strains in both pBR325 and pBR235 derivatives, as observed previously for various *E. coli* strains containing pBR325 [52,55]. The Hfq deficient strains contained a higher copy number per cell, in agreement with previous observations [58]. Consequently, the mutation rates per plasmid for the Hfq deficient strains should be even lower. 

### 2.3. Statistical Analysis 

Mutation rate estimates were calculated using the rSalvador package [59]. To compare multiple rates, likelihood ratio tests were performed (similar to that described in [60]) and false discovery rates (FDR) were calculated [61] as this allows preservation of high statistical powers (e.g., in comparison with Bonferroni corrections). To visually identify possible statistical differences, 84% (instead of 95%) confidence intervals were used [62]. 

### 2.4. Binding Assays of Hfq-CTR on d(G_3_T)_4_

The peptide corresponding to CTR domain of Hfq (residues 64 to 102, referred to as Hfq-CTR) was synthetized by Proteogenix SA (France). Full length and ∆CTR forms of the proteins were purified as described previously [63]. The sequence of the Hfq-CTR peptide is SRPVSHHSNNAGGGTSSNYHHGSSAQNTSAQQDSEETE. The oligonucleotide d(G_3_T)_4_ was purchased from Eurogentec. The G-quadruplexes were prepared in water by heating at 95 °C for 5 min and then slowly cooling to room temperature. We confirmed using Synchrotron Radiation Circular Dichroism (SRCD) that the quadruplex forms, even without salts. The binding of Hfq-CTR to d(G_3_T)_4_ was investigated with a gel shift assay (EMSA). d(G_3_T)_4_ was incubated with Hfq-CTR at room temperature for 20 min. Band shifts were resolved on non-denaturing gradient 4–20% polyacrylamide gel. The native gel was run for 2 h at room temperature with 40 mM Tris-Acetate, 1 mM ethylenediaminetetraacetic acid, pH 8.0 (TAE) buffer, stained with ethidium bromide nucleic acid stain and imaged with a G-BOX system (Syngene, Cambridge, UK). For SRCD analysis, measurements and data collection were carried out on DISCO beam-line at the SOLEIL Synchrotron (proposal 20190015) [64]. Then, 2 µl of samples were loaded into circular demountable CaF_2_ cells of 50 microns path length [65]. Three separate data collections with fresh sample preparations were carried out to ensure consistency and repeatability. Spectral acquisitions of 1 nm steps at 1.2 s integration time, between 320 and 170 nm, were performed in triplicate for the samples as well as for the baselines. (+)-camphor-10-sulfonic acid (CSA) was used to calibrate amplitudes and wavelength positions of the SRCD experiment. Data-analyses including averaging, baseline subtraction, smoothing and scaling were carried out with CDtool [66]. SRCD units were kept in mdeg, because normalization to obtain Δε units is not convenient for DNA and peptide complexes. The same cell and the same samples concentrations were used for all loadings.

## 3. Results

### 3.1. Influence of Hfq and the Hfq-CTR on the Instability of Quadruplex-Forming Repeats 

**Figure 1:**
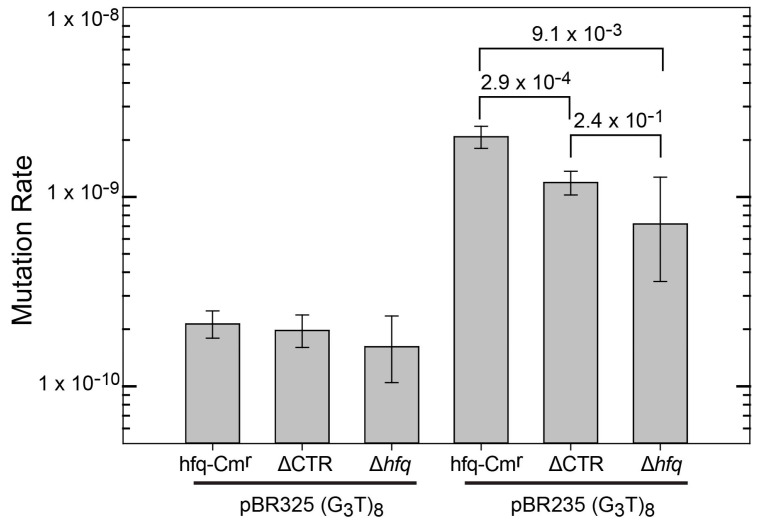
Mutation rates for (G_3_T)_8_ repeats in plasmids pBR325 and pBR235 in MG1655 *hfq*-Cm^r^ (reference strain), MG1655 HfqNTR72-Cm^r^ (∆CTR), and MG1655 *hfq*::Cm^r^ (∆*hfq*) [38]. Mutation rates were determined as described under Materials and Methods. Data for MG1655 *hfq*-Cm^r^ and MG1655 NTR72-Cm^r^ with both pBR325 and pBR235 represent results from two independent Luria–Delbrück fluctuation analyses. Results for plasmids in MG1655 *Δhfq*::Cm^r^ represent a single Luria–Delbrück fluctuation analysis. Error bars represent 84% confidence intervals. Numbers represent false discovery rates.

A set of hfq mutants was tested to determine if the 72 amino acid N-terminal domain or the 38 amino acid C-terminal domain is responsible for interacting with G-quadruplex DNA in cells. For this, plasmids were inserted into three MG1655 derivatives containing cassettes inserted in the hfq locus. One cassette contained a wild-type hfq gene (MG1655 *hfq*-Cm^r^), another the region of the gene encoding only the first 72 amino acids of Hfq (NTR72, referred to as ∆CTR), and one with only the *CAT* gene resulting in loss of Hfq (termed ∆*hfq*). Expression of Hfq-CTR in vivo was not possible as the CTR domain produced in the cell is unstable [47]. Mutation rates for the (G_3_T)_8_ repeat are shown in Figure 1. Plasmids containing (G_3_T)_8_ were used as they exhibit a higher mutation rate than (G_3_T)_4_ that was used for the in vitro binding analysis (Figure S1). In the pBR325 orientation, in which the transcription and replication machinery collide, mutation rates were extremely low and little difference was observed in mutation rates between the hfq^+^ and ∆CTR strains. The ∆*hfq* strain had a similar mutation rate, albeit with a plasmid higher copy number. The low rates in the pBR325 strains may reflect a minimal or negligible rate of G-quadruplex formation in this plasmid. The strains containing the (G_3_T)_8_ repeat in pBR235 exhibited higher mutation rates and statistically significant differences were observed in mutation rates among the three different genetic backgrounds. Compared with the hfq^+^ strain, both the ∆CTR and ∆*hfq* strains exhibited statistically significant lower mutation rates. Rates were not statistically different between the ∆CTR and ∆hfq strains. This can be attributed to the large confidence intervals of the ∆*hfq* strain, resulting from the lower number of plates used (36 for both the hfq^+^ and ∆CTR strains and 16 for the ∆*hfq* strain, respectively). Results are consistent with an interpretation that G-quadruplex stabilization does not occur in the absence of full-length Hfq or the CTR domain. These results suggest that the propensity for binding G-quadruplex DNA was retained in the C-terminal domain of the Hfq protein. 

### 3.2. Interaction of Hfq-CTR with G-Quadruplex DNA

**Figure 2:**
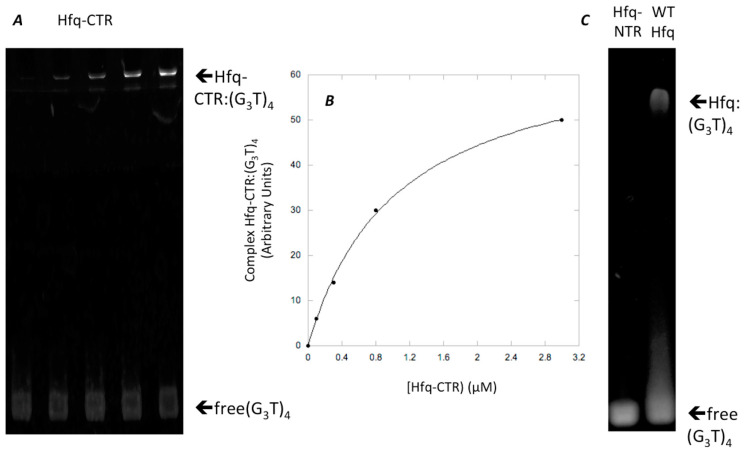
Hfq Binding to (G_3_T)_4_. (**A**) Hfq-CTR binding to (G_3_T)_4_, (G_3_T)_4_ concentration 0.1 μM, while Hfq-CTR concentration ranged from 0 to 3 μM. (**B**) Graphic analysis of Hfq-CTR binding to (G_3_T)_4_ shown in A. (**C**) controls: Hfq-NTR and wild type Hfq binding to (G_3_T)_4_.

**Figure 3:**
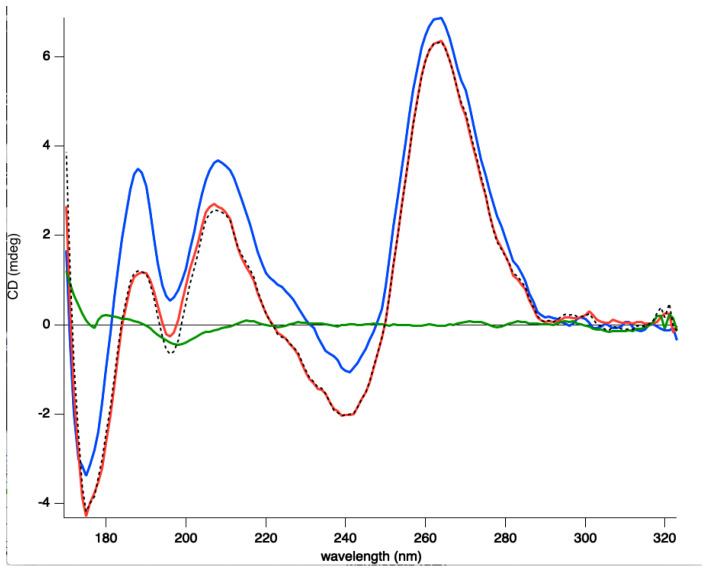
Synchrotron radiation circular dichroism (SRCD) analysis of the (G_3_T)_4_ quadruplex complexed to Hfq-CTR. Spectra of (G_3_T)_4_ in the absence (red) and presence of Hfq-CTR (blue). Hfq alone (green). The spectrum of the complex (blue) is similar to the sum of the (G_3_T)_4_ and Hfq-CTR spectra (dotted black), differing only in the strength of its amplitudes. This signifies most likely that upon complex formation, an enhancement of already existing structural features in the quadruplex is occurring. It is not clear whether the Hfq-CTR in contact with (G_3_T)_4_ changes its structure, to form amyloids, which would change the 210–220 nm amplitudes of the spectrum [38].

While full length Hfq binds to DNA quadruplexes [43], Hfq-CTR binding to DNA-quadruplex has not been investigated. Hfq:d(G_3_T)_4_ quadruplex complex formation was confirmed by EMSA and the equilibrium dissociation constant (K_d_) of the complex was 1059 ± 74 nM (Figure 2). This value was similar to that of full length Hfq for parallel quadruplexes [43]. SRCD spectroscopy was then used to see how the protein affects G-quadruplex structures. The (G_3_T)_4_ quadruplex alone produced a typical spectrum in accordance with previously reported parallel quadruplex CD spectra [67,68,69]. Specifically, we observed a peak conservation for the ~264 nm (maximum) and ~245 nm (minimum) as well as a positive one at ~205 and ~185 nm and a negative one below 180 nm (minimum). For the spectra of the (G_3_T)_4_ Hfq-CTR mixture, stronger amplitudes in the same positions without significant changes in the maxima and minima were observed. As shown in Figure 3, in the region from 320–200 nm where the CD contributions originate from the nucleotide bases, sugars, and phosphates in a general way, with no particular changes, such as CD signal inversions or peak shifts were observed for the complex in comparison with the (G_3_T)_4_. This signifies that the overall quadruplex structure has been preserved and rather reinforced its spectral amplitudes. The increases of the amplitudes around 189 and 263 nm are most likely a result of increased G-G stacking and Hoogsteen base pairing, respectively [69].

Note that our previous analysis used a dG_7_ sequence [43], while our current analysis uses a d(G_3_T)_4_ sequence instead because it is closer to natural sequences found in the genome. Nevertheless, we ensure that both sequences form parallel quadruplexes (see Figure S2), that the affinity of Hfq-CTR is similar for d(G_3_T)_4_ and dG_7_ (K_d_ = 1059 ± 74 nM for (G_3_T)_4_ vs. K_d_ = 981 ± 96 nM for dG_7_) and that Hfq-CTR reinforces the structure of d(G_3_T)_4_ and dG_7_, especially in the base-paring region around 260 nm (Figure S2).

## 4. Discussion 

*The* Hfq-CTR increases stability of G-quadruplex repeats—Hfq is a post-transcriptional regulator, which influences RNA structure and RNA-based regulations [41]. However, it also binds to DNA [35,40], including regions of DNA bending and, as shown previously, Hfq binds to G-quadruplex [43]. To understand which domain of Hfq binds to G-quadruplex in cells, the influence of the Hfq protein and the Hfq-NTR on G-quadruplex instability was analyzed. For the (G_3_T)_8_ repeat, mutation rates were reduced in the ∆CTR and ∆*hfq* strains in pBR235 but not pBR325 in the MG1655 background. These results are consistent with an interpretation that, in wild type cells, Hfq binds and stabilizes the quadruplex formed in the (G_3_T)_8_ repeat, in accordance with its ability to help nucleic acid annealing [70], and this could favor structure accumulation and increase the rate of deletion mutagenesis. This effect is reduced in the strain containing the 72 amino acid N-terminal domain, supporting the conclusion that the Hfq-CTR is responsible for binding, as confirmed by EMSA and the SRCD results (Figure 2 and Figure 3). Note that Hfq-CTR is highly variable in length and sequence composition across species. For example, it is smaller or absent in some Gram-positive bacteria, where its function remains elusive [71]. Possibly another protein could play a similar function in genome instability in these bacteria. Additionally, some of these bacteria such as *Bacillus subtilis* or *Clostridium difficile* are low G + C content bacteria. Whether the size of Hfq-CTR could be related to bacteria G + C content and presence of quadruplexes should be analyzed further.

**Figure 4:**
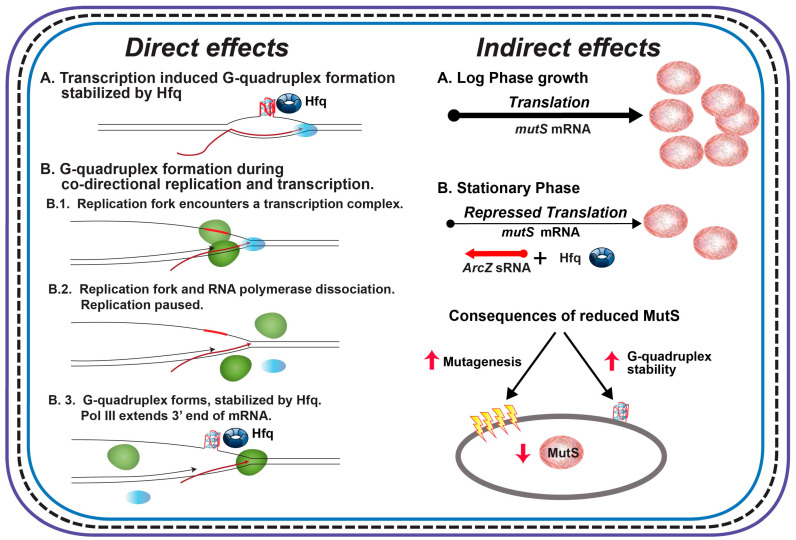
Model of direct and indirect effects of Hfq on genome instability. Description of the figure is included in the text. Direct effects: Green ovals represent DNA Pol III; blue ovals, RNA polymerase. Hfq is represented by the donut. Indirect effects: MutS is represented by the brown ovals. Red arrows represent increased or decreased levels.

Our interpretation of the extant results is that the (G_3_T)_8_ repeat in the *tet* gene in pBR325 forms G-quadruplex structures at a very low level and that stabilization by Hfq does not lead to a measurable stabilization of structures. On the other hand, the higher rates in pBR235 suggest increased structure formation resulting from the co-directional movement of transcription and replication. As DNA replication occurs at 530–750 nt/sec [72] and transcription 12–24 nt/sec [73], the replication fork will encroach on the transcription complex. When this happens, DNA replication pauses, the RNA polymerase is displaced, and the mRNA is used as a template to restore the replication fork [74,75]. Thus, co-directional collision during transcription of the (G_3_T)_8_ repeat may promote G-quadruplex formation in this plasmid by providing a time window during replication restart for structure formation to occur (Figure 4, Direct Effects). The replisome displaces RNA polymerase and the mRNA during head-on collisions in pBR325 [74] and this must not lead to an increased opportunity for structure formation.

The lack of an effect in inactivation of Hfq or deletion of the CTR domain in the pBR325 plasmid presumably reflects a very minimal level of formation of the G-quadruplex in this strain. Overall, mutation rates in the MG1655 background were lower than observed previously in *E. coli* BW24113 or MC4100 backgrounds [43]. When cloned into the *CAT* gene in an orientation in which transcription could drive G-quadruplex formation ((G_3_T) repeats comprise the nontemplate strand), mutation rates for the (G_3_T)_8_ repeat were increased by factors of 350 and 270 for pBR325 and pBR235, respectively, compared with an orientation in which transcription-induced G-quadruplex formation was not possible ((AC_3_) repeats comprise the nontemplate strand) [43]. The mutation rates without transcription-induced G-quadruplex formation were approximately 2–4 × 10^−8^, reflecting a possible basal rate in the *CAT* gene in this strain (see sup Figure S1). When cloned in the *tet* gene, as analyzed here, mutation rates in an MC4100 background, including a ∆hfq strain were much lower than observed for the *CAT* gene, with the ∆*hfq* mutation rate at 2 × 10^−9^, similar to that observed here with the pBR325 plasmid. Differences in rates in different genetic backgrounds and in plasmids with different directions of the replication fork have been observed and discussed in detail previously [43]. The lower rate of (G_3_T)_8_ deletion observed here reflects several factors including the local sequence environment and the characteristics of transcription that can influence G-quadruplex structure formation and thus, the subsequent probability of deletion. In addition, mutation to a Tet^r^ phenotype requires complete deletion of the (G_3_T)_8_ insert and one flanking BamHI direct repeat from the *tet* gene, while partial or complete deletion can restore a Cm^r^ phenotype in the *CAT* gene [43]. Thus, this minimal rate in pBR325 plasmids likely reflects a situation where G-quadruplex formation by transcription is not occurring at any appreciable level. 

A previous observation of an increased plasmid copy number in a strain deficient for Hfq [58] was confirmed in this analysis. Results strongly argue that the Hfq-CTR domain is responsible for G-quadruplex stabilization and an increased mutation rate. It is also of interest that an increase in copy number does not proportionally increase mutation rates in the Δ*hfq* strains. This suggests that other pleiotropic consequences of the absence of Hfq are responsible. This may include alteration of concentrations of proteins involved in replication and repair of aberrant DNA conformations or selective loss of plasmids involved in the deletion process. Alterations in DNA repair and double strand break repair have been reported in Hfq deficient strains and strains deficient in sRNAs that interact with Hfq [76,77,78,79].

Given the results in this and a previous paper [43], the following observations regarding pathways for G-quadruplex formation in cells and the effects of Hfq on G-quadruplex instability are worth noting. First, in the context of the *CAT* gene, when the (G_3_T)_8_ repeat comprises the nontemplate strand, G-quadruplex can form during transcription via R-loop formation (Figure 4, Direct Effects, A.) and is stabilized by Hfq binding leading to an increased rate of repeat deletion. Analysis of revertants [43] suggests that deletions likely occur by replication slippage involving template misalignment between either flanking direct repeats (EcoRI sites) or (G_3_T) repeats, both mediated by DNA secondary structure formation [52,53,54,55,80,81,82,83]. Second, the apparent lack of transcription-induced G-quadruplex formation for the (G_3_T)_8_ repeat in the *tet* gene reveals that the ability of transcription to drive G-quadruplex depends on sequence context and/or the characteristics of transcription. It is noted that rates of transcription can vary greatly for different genes in *E. coli* [73]. Third, in the *tet* gene, when transcription induced G-quadruplex formation does not occur, the rate of deletion, and presumably G-quadruplex structure formation, depends on the directions of replication and transcription [74,75]. When a replication fork and transcription complex collide co-directionally, the replication fork displaces RNA polymerase and dissociates leaving the end of the mRNA to serve as a 3′ primer for replication restart. This pause and the RNA-DNA hybrid when including the (G_3_T)_8_ repeat allows for G-quadruplex formation that can be stabilized by Hfq (Figure 4, Direct Effects, B.). Fourth, as discussed previously [43], Hfq can also have other indirect effects (Figure 3, Indirect Effects). In stationary phase, Hfq levels increase and Hfq interacts with ArcZ sRNA to repress *mutS* transcription [84]. This leads to an increase in overall mutagenesis from a reduction in the capacity for mismatch repair. Note that large gaps opened up during mismatch repair [85] may also provide opportunities for G-quadruplex formation. It is also noted that MutS can bind to G-quadruplex structures [34] and may also afford a level of stabilization leading to increased deletion. Finally, other indirect effects may be due to sRNA-based regulation of some transcription factors [86] or of other nucleoid proteins that could influence quadruplex stability [87,88].

In conclusion, while Hfq CTR function has remained enigmatic for years [41], it is now clear that it exhibits a plethora of binding affinities for nucleic acids, and plays a pivotal role in DNA mechanical properties, transcription, genome instability, and mutation in cells [38,39,89,90]. 

## Supplementary Materials

The following are available online at https://www.mdpi.com/2076-2607/8/10/1598/s1, Figure S1: Mutation rates for (G_3_T)_n_ and (C_2_A)_n_ repeats in plasmid pBR325 in BW25113. Figure S2: SRCD spectra of Hfq-CTR with dG_7_.
