# Supplementary material for: Crucial Role of the C-Terminal Domain of Hfq Protein in Genomic Instability
Source: Microorganisms. 2020 Oct 17;8(10):1598. doi: 10.3390/microorganisms8101598 (PMC7603069; doi:10.3390/microorganisms8101598)
Supplement: Supplementary file 1 [file microorganisms-08-01598-s001.pdf]

microorganisms

Crucial role of the C-terminal domain of Hfq protein in genomic instability

Virali J. Parekh[1], Frank Wien[2], Wilfried Grange[3,4], Thomas A. De Long[1], Véronique Arluison[4,5]* and Richard R. Sinden[1]*

SUPPLEMENTAL INFORMATION

Figures

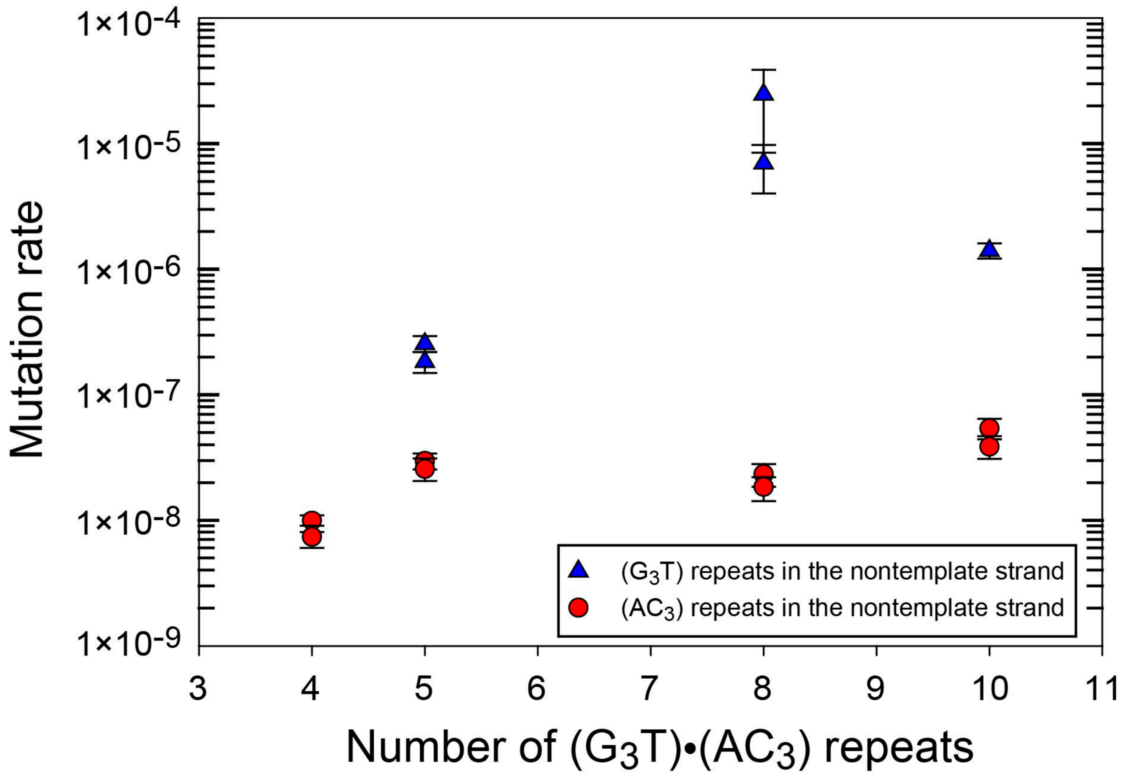

Figure S1: Effect of length and repeat orientation of $(G_3T)\bullet(AC_3)_n$ repeats cloned in the *CAT* gene in pBR325 in *E. coli* BW25113.

Mutation rates were measured for plasmids containing $(G_3T)_n$ or $(AC_3)_n$ repeats cloned in the nontemplate strand. Mutation rates were determined by Luria-Delbrück fluctuation assays [1] and calculated using FluCalc [2], as described in a previous publication [3]. This figure confirms that when $(G_3T)_n$ comprised the nontemplate strand (▲), transcription can drive formation of G-quadruplex structures leading to high mutation rates (to >10^{-5} in this strain) [3]. Moreover, the mutation rate increases with increasing lengths of $(G_3T)_n$ repeats. Lower mutation rates (10^{-8} - <10^{-7}) were observed when $(AC_3)_n$ repeats comprised the nontemplate strand (●). This may reflect a minimal or basal rate of G-quadruplex formation. As G-quadruplex is not driven by transcription, it may form at low levels in the leading or lagging strands during DNA replication. Alternatively,

misalignment during replication slippage may occur in the absence of G-quadruplex structure formation.

The figure also illustrates that longer $(G_3T)_n$ repeats exhibit a higher mutation rate than shorter repeats, as has been observed for deletion of other repeats [4-6], although one does not necessarily expect a linear relationship. In fact, this can be complicated as reversion to a Cm^r phenotype can occur by partial or complete deletion [3-5]. The rate of reversion to a Cm^r phenotype will depend on both the rate of structure formation and then the probability of mutation event. Cm^r reversion occurs by restoration of the reading frame by either complete deletion of the insert and one copy of the restriction site, or by partial deletion that restores the reading frame. This is believed to occur by slipped misalignment during replication [5]. $(G_3T)_{10}$ may have a lower mutation rate than $(G_3T)_8$ due to structure formation that reduces misalignment between flanking direct repeats or that leads to nonselectable events that do not restore the reading frame.

The data point for $(G_3T)_{10}$ represents combined data for two experiments. Other data points represent individual Luria-Delbrück fluctuation assays. Error bars represent 0.95 confidence intervals.

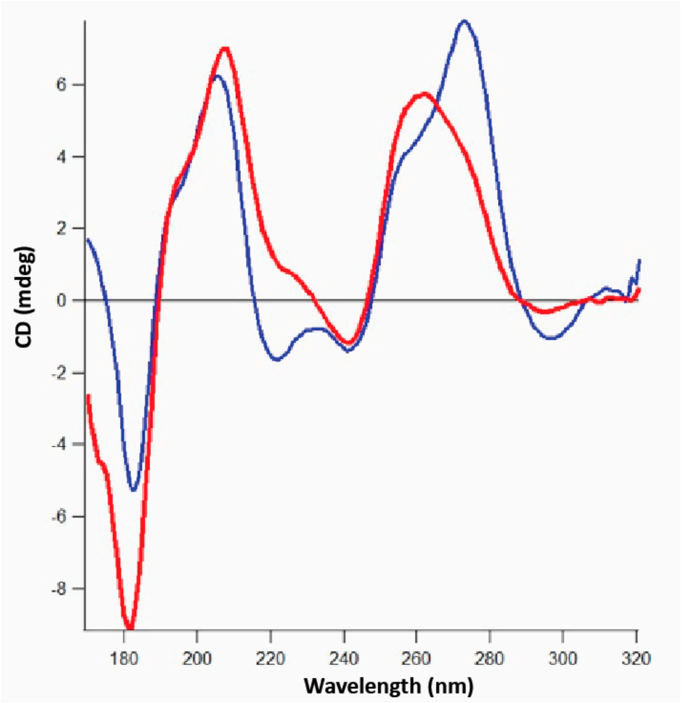

Figure S2. SRCD analysis of the dG_7 quadruplex complexed to Hfq-CTR by SRCD spectroscopy. Spectra of dG_7 in the absence (red) and presence of Hfq-CTR (blue). The peak conservation for the ~265 nm (Maximum) and ~245 nm (Minimum) as well as a positive one at ~205 nm and ~185 nm and a negative one below 180 nm (Minimum) confirms that dG_7 forms a parallel quadruplex, as $d(G_3T)_4$. The increase in the ~265 nm region signifies that upon complex formation, an enhancement of already existing structural features (base pairing and base-stacking) in the quadruplex is occurring. The possible structural change of Hfq-CTR structure in

contact with dG_7, which would change the 200-220 nm amplitudes of the spectrum [7], makes the quantitative analysis of this region more difficult.

References

1. Luria, S.E.; Delbruck, M. Mutations of Bacteria from Virus Sensitivity to Virus Resistance. *Genetics* **1943**, *28*, 491-511.
2. Radchenko, E.A.; McGinty, R.J.; Aksenova, A.Y.; Neil, A.J.; Mirkin, S.M. Quantitative Analysis of the Rates for Repeat-Mediated Genome Instability in a Yeast Experimental System. *Methods in molecular biology (Clifton, N.J.)* **2018**, *1672*, 421-438, doi:10.1007/978-1-4939-7306-4_29.
3. Parekh, V.J.; Niccum, B.A.; Shah, R.; Rivera, M.A.; Novak, M.J.; Geinguenaud, F.; Wien, F.; Arluison, V.; Sinden, R.R. Role of Hfq in Genome Evolution: Instability of G-Quadruplex Sequences in E. coli. *Microorganisms* **2019**, *8*, doi:10.3390/microorganisms8010028.
4. Edwards, S.F.; Hashem, V.I.; Klysik, E.A.; Sinden, R.R. Genetic instabilities of (CCTG).(CAGG) and (ATTCT).(AGAAT) disease-associated repeats reveal multiple pathways for repeat deletion. *Molecular carcinogenesis* **2009**, *48*, 336-349, doi:10.1002/mc.20534.
5. Hashem, V.I.; Rosche, W.A.; Sinden, R.R. Genetic assays for measuring rates of (CAG).(CTG) repeat instability in Escherichia coli. *Mutation research* **2002**, *502*, 25-37, doi:10.1016/s0027-5107(02)00026-x.
6. Iyer, R.R.; Pluciennik, A.; Rosche, W.A.; Sinden, R.R.; Wells, R.D. DNA polymerase III proofreading mutants enhance the expansion and deletion of triplet repeat sequences in Escherichia coli. *The Journal of biological chemistry* **2000**, *275*, 2174-2184, doi:10.1074/jbc.275.3.2174.
7. Malabirade, A.; Partouche, D.; El Hamoui, O.; Turbant, F.; Geinguenaud, F.; Recouvreux, P.; Bizien, T.; Busi, F.; Wien, F.; Arluison, V. Revised role for Hfq bacterial regulator on DNA topology. *Scientific reports* **2018**, *8*, 16792, doi:10.1038/s41598-018-35060-9.
